# Neurovascular unit uncoupling in diabetic retinopathy: molecular mechanisms and stage-adapted therapeutic strategies

**DOI:** 10.3389/fendo.2026.1896091

**Published:** 2026-07-01

**Authors:** Jing Chen, Ling Zhang

**Affiliations:** Department of Ophthalmology, People’s Hospital of Leshan, Leshan, Sichuan, China

**Keywords:** blood-retinal barrier, diabetes complications, diabetic retinopathy, neuroinflammation, neurovascular uncoupling, neurovascular unit, oxidative stress, stage-adapted therapy

## Abstract

Diabetic retinopathy (DR) is a major neurovascular complication of diabetes and remains a leading cause of vision loss among working-age adults worldwide. Although DR has traditionally been classified as a microvascular complication, it is now increasingly recognized as a neurovascular degenerative disorder involving coordinated injury to neuronal, glial, vascular, and extracellular matrix components of the retinal neurovascular unit (NVU). The NVU provides the structural and functional basis for coupling neuronal activity to local blood flow and for maintaining retinal immune and barrier homeostasis. In diabetes, chronic hyperglycemia, oxidative stress, inflammation, metabolic dysregulation, impaired vascular endothelial growth factor (VEGF)/angiopoietin-Tie (Ang/Tie) signaling, abnormal intercellular communication, and epigenetic memory progressively disrupt the coordinated interactions among NVU components, leading to neurovascular uncoupling. This concept helps explain why retinal functional abnormalities and neurodegenerative changes may precede clinically visible vascular lesions. In this review, we summarize cell-specific NVU alterations and the molecular mechanisms that drive neurovascular uncoupling in DR. We also discuss how this framework may support earlier diagnosis, mechanism-based phenotyping, and stage-adapted treatment strategies. Established therapies, including anti-vascular endothelial growth factor (anti-VEGF) agents, corticosteroids, and angiopoietin-2 (Ang-2)/Tie-2-directed vascular stabilization, are considered together with investigational approaches targeting oxidative stress, inflammation, neuroprotection, metabolic reprogramming, epigenetic regulation, and drug delivery. Reframing DR as a diabetes-driven disorder of early NVU uncoupling may help shift clinical thinking from late vascular rescue toward mechanism-based neurovascular protection, precision phenotyping, and stage-adapted intervention.

## 1 Introduction

Diabetic retinopathy (DR) affects more than 100 million individuals worldwide, and its global burden is expected to continue increasing with the rising prevalence of diabetes (1). As a diabetes-related neurovascular complication, DR reflects not only local retinal injury but also the cumulative effects of systemic metabolic dysregulation, inflammatory activation, vascular instability, and impaired cellular communication. For decades, DR has been defined, staged, and treated primarily as a microvascular complication of diabetes. Current clinical classification systems are largely based on visible vascular lesions, including microaneurysms, hemorrhages, venous beading, intraretinal microvascular abnormalities, macular edema, and retinal neovascularization (2). This vascular-centered framework has been essential for screening, staging, and treatment decision-making. However, it does not fully explain the early functional abnormalities, retinal neurodegeneration, and variable disease progression observed in many patients with diabetes. Accumulating evidence over the past two decades has shown that retinal neuronal dysfunction and neurodegeneration are not merely secondary consequences of advanced vascular disease, but may occur early in the course of DR, sometimes before clinically detectable microvascular abnormalities (3). Functional abnormalities, including reduced contrast sensitivity, impaired dark adaptation, color vision defects, and abnormal electroretinographic responses, have been reported in patients with diabetes even in the absence of overt retinopathy (4). Structural changes, such as thinning of the retinal nerve fiber layer and ganglion cell complex, further support the presence of early neuronal injury (5). These findings suggest that DR should not be viewed solely as a vascular disease, but rather as a disorder involving coordinated injury to neural, glial, and vascular compartments.

The concept of the retinal neurovascular unit (NVU) provides an integrative framework for understanding this broader pathogenesis. The NVU consists of retinal neurons, including photoreceptors, bipolar cells, amacrine cells, and ganglion cells; glial cells, including Müller cells, astrocytes, and microglia; vascular cells, including endothelial cells and pericytes; and the surrounding extracellular matrix (6, 7). These components interact through direct cell contact, paracrine signaling, metabolic exchange, and extracellular matrix-mediated communication. Together, they coordinate neuronal activity with local blood flow, preserve the integrity of the blood-retinal barrier (BRB), regulate immune surveillance, and maintain retinal metabolic homeostasis. Under physiological conditions, neurovascular coupling allows increases in neuronal activity to be matched by corresponding increases in local perfusion, thereby ensuring adequate oxygen and nutrient delivery to metabolically active retinal tissue (8). This process requires intact neuronal signaling, glial transduction, endothelial responsiveness, pericyte-mediated capillary regulation, and appropriate vasoactive mediator release. In the diabetic retina, however, chronic hyperglycemia, advanced glycation end products, mitochondrial dysfunction, oxidative stress, inflammatory cytokines, vascular endothelial growth factor (VEGF)/angiopoietin-Tie (Ang/Tie) imbalance, and epigenetic alterations progressively disturb these coordinated interactions (9, 10). The result is neurovascular uncoupling, characterized by impaired neuronal activity-dependent vascular regulation, glial dysfunction, vascular barrier failure, and chronic inflammatory activation.

This NVU-based concept has important clinical implications (11). First, it may explain why retinal dysfunction can be detected before visible vascular lesions appear, suggesting that early DR may begin as a functional and molecular disorder rather than a purely structural vascular disease. Second, it provides a rationale for earlier diagnostic strategies using electrophysiology, optical coherence tomography (OCT), optical coherence tomography angiography (OCTA), functional imaging, and molecular biomarkers to identify subclinical NVU impairment. Third, it supports a mechanism-based approach to treatment, in which patients may be stratified according to dominant pathogenic features such as neurodegeneration, inflammation, vascular leakage, ischemia, or metabolic vulnerability. Finally, it suggests that future therapy should not be limited to late-stage vascular rescue, but should also aim to preserve neuronal function, restore glial homeostasis, stabilize the BRB, and maintain intercellular communication within the NVU.

Previous reviews have discussed retinal neurodegeneration, vascular dysfunction, inflammation, or metabolic injury in DR as separate pathological processes. In contrast, this review places NVU uncoupling at the center of diabetes-related retinal injury. It integrates cell-specific alterations, metabolic and inflammatory mechanisms, intercellular signaling defects, early diagnostic opportunities, and stage-adapted treatment strategies into a translational framework. We first summarize the physiological structure and function of the retinal NVU. We then discuss pathological changes in NVU components and analyze the molecular mechanisms that drive neurovascular uncoupling, including metabolic dysregulation, oxidative stress, neuroinflammation, disrupted VEGF/Ang-Tie and neurotrophic signaling, epigenetic memory, and abnormal intercellular communication. Finally, we evaluate current and emerging therapeutic strategies according to their relevance to NVU restoration and clinical translation. By reframing DR as a disorder of early NVU uncoupling, this review aims to support a shift from isolated vascular treatment toward earlier, integrated, and more precise neurovascular protection.

### 1.1 Literature search and selection criteria

This narrative review was guided by the Scale for the Assessment of Narrative Review Articles (SANRA) framework (12). A structured literature search was conducted in PubMed, Web of Science, and Embase to identify peer-reviewed articles published between January 2000 and May 2026. Earlier landmark studies were also included when they provided foundational evidence for diabetic retinopathy, neurovascular coupling, metabolic memory, or diabetes-related retinal complications. Priority was given to systematic reviews, meta-analyses, randomized controlled trials, large clinical studies, and mechanistic studies directly relevant to retinal NVU dysfunction, diabetes-induced metabolic injury, inflammatory signaling, vascular instability, and therapeutic targeting. Because this was not a systematic review, formal risk-of-bias assessment and quantitative evidence synthesis were not performed.

## 2 Physiological organization and coupling function of the retinal neurovascular unit

### 2.1 Structural components of the NVU

The retinal NVU represents a highly organized functional unit, and its structural integrity is fundamental to maintaining normal retinal physiology (6). Neuronal components include photoreceptors (rods and cones), bipolar cells, amacrine cells, horizontal cells, and ganglion cells, which together form the neural circuitry for visual signal transduction. Among glial components, Müller cells are the main retinal glial cells. They span the entire retinal thickness, and their processes ensheathe both neurons and blood vessels to provide structural support, metabolic maintenance, and signal modulation (13). Astrocytes are located mainly in the nerve fiber layer, where their endfeet envelope retinal vessels to regulate vascular tone and maintain the BRB. Microglia are resident immune cells that become activated under pathological conditions and participate in immune surveillance (14).

Vascular components include endothelial cells, pericytes, and vascular smooth muscle cells. Retinal endothelial cells form the inner BRB via tight junctions, which strictly control molecular exchange between the blood and retinal tissue (15). Pericytes share a basement membrane with endothelial cells and regulate vascular tone, angiogenesis, and BRB integrity through direct physical contact and paracrine signaling (16, 17). Notably, the retina has one of the highest pericyte-to-endothelial cell ratios among vascularized tissues, reaching 1:1 to 1:3. This ratio significantly exceeds that of other tissues and highlights the high precision required for retinal vascular regulation (18, 19).

### 2.2 Physiological mechanisms of neurovascular coupling

Neurovascular coupling refers to the process of dynamic local blood flow regulation in response to neuronal activity, ensuring that metabolically active neural tissue receives adequate oxygen and nutrients (8). This process involves a complex intercellular signaling network. Glutamate released from neurons acts upon Müller cells and astrocytes, stimulating these glial cells to generate vasoactive mediators. Glial endfeet, which are in direct contact with vessels, then transduce these neuronal signals into vascular regulatory responses. Major vasodilatory factors include nitric oxide (NO), prostaglandin E2 (PGE2), adenosine, and potassium ions (7). Among these, NO derived from neuronal (nNOS) and endothelial (eNOS) nitric oxide synthases serves as a critical vasodilator. The precise balance between vasoconstrictive signals, such as 20-hydroxyeicosatetraenoic acid (20-HETE), endothelin-1, and angiotensin II, and vasodilatory signals ensures an accurate match between blood supply and metabolic demand.

### 2.3 Maintenance of the blood-retinal barrier

The BRB consists of inner and outer barriers, corresponding to the tight junctions between retinal vascular endothelial cells and those between retinal pigment epithelial (RPE) cells, respectively (15). The inner barrier is a critical function of the NVU. Its integrity depends on the proper expression and localization of endothelial tight junction proteins, including occludin, claudin-5, and zonula occludens-1 (ZO-1), as well as the support provided by pericytes and glial cells (9).

**Figure 1:**
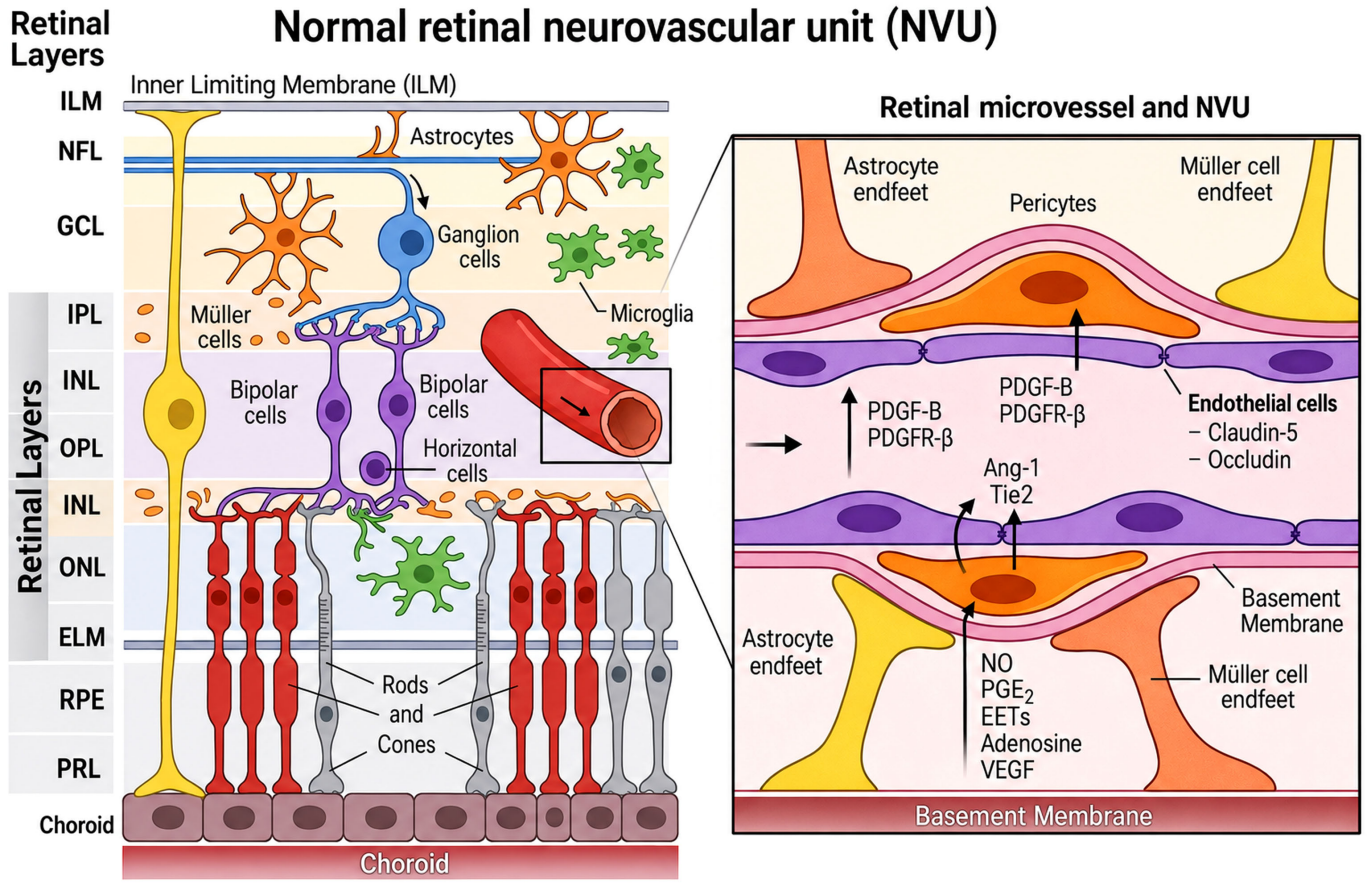
Structural organization and physiological functions of the retinal neurovascular unit. The retinal neurovascular unit is composed of neurons, including ganglion cells, bipolar cells, and photoreceptors; glial cells, including Müller cells, astrocytes, and microglia; and vascular cells, including endothelial cells and pericytes. The enlarged retinal microvessel panel illustrates the close spatial relationship among endothelial cells, pericytes, astrocyte endfeet, Müller cell endfeet, and the basement membrane. These cellular components interact through coordinated molecular signaling networks to maintain retinal homeostasis, neurovascular coupling, and inner blood-retinal barrier integrity. In the schematic, green cells denote microglia, yellow structures denote Müller cells and Müller cell endfeet, orange structures denote astrocytes and pericytes according to location, and purple vascular lining denotes endothelial cells.

Pericytes secrete angiopoietin-1 (Ang-1), which activates Tie-2 receptors on endothelial cells to promote endothelial cell survival and tight junction assembly (16). Other factors, including transforming growth factor-β (TGF-β) and platelet-derived growth factor-B (PDGF-B), participate in pericyte recruitment and BRB stabilization (17, 18). Müller cell and astrocyte endfeet wrap around blood vessels and release glial cell-derived neurotrophic factor (GDNF) and pigment epithelium-derived factor (PEDF), which support BRB function (13). The structural organization and physiological functions of the retinal NVU are illustrated in **Figure 1**.

## 3 Cell-specific NVU alterations under diabetic stress

### 3.1 Neuronal injury and dysfunction

Retinal neuronal injury is increasingly recognized as an early event in DR, sometimes preceding clinically detectable microvascular lesions (3). Functional studies have shown that patients with diabetes may develop reduced multifocal electroretinography amplitudes and prolonged implicit times before obvious funduscopic abnormalities become apparent, indicating early impairment of retinal neural activity (20). Structural imaging studies have also demonstrated thinning of the retinal nerve fiber layer and ganglion cell complex in patients with diabetes, supporting the concept that neurodegeneration contributes to early retinal dysfunction (4).

Experimental studies further indicate that retinal ganglion cells are particularly vulnerable to the diabetic milieu. Increased ganglion cell apoptosis, impaired synaptic transmission, and reduced expression of synaptic proteins have been reported in diabetic animal models (21). Photoreceptor and bipolar cell dysfunction may also occur, leading to abnormal glutamate handling and impaired signal transmission within the inner and outer retinal networks (22). These changes provide a structural and functional basis for subtle visual deficits that may arise before advanced vascular complications develop.

Importantly, neuronal dysfunction does not remain an isolated neural event. Because neuronal activity normally initiates activity-dependent vascular responses, impaired neuronal signaling can weaken the physiological trigger for neurovascular coupling. Thus, early neuronal injury may reduce the ability of the retina to match local perfusion with metabolic demand, thereby contributing to the initial stage of NVU uncoupling in DR.

### 3.2 Glial activation and dysfunction

Glial cells are central regulators of retinal homeostasis and serve as critical intermediaries between neurons and the vasculature. In DR, glial activation may initially represent an adaptive response to metabolic stress; however, sustained activation gradually becomes maladaptive and promotes neuroinflammation, excitotoxicity, and BRB disruption (23–25). Müller glial cells are key responders to diabetic retinal stress. They span the entire retinal thickness and are anatomically positioned to communicate with neurons, vessels, and the vitreoretinal interface (13). Under diabetic conditions, Müller cells undergo reactive gliosis, characterized by increased glial fibrillary acidic protein expression, cellular hypertrophy, and altered expression of ion and water channels (25, 26). Reduced expression or function of glutamate transporters, including glutamate-aspartate transporter (GLAST) and glutamate transporter-1 (GLT-1), impairs glutamate clearance and may contribute to excitotoxic neuronal injury. In parallel, abnormal Kir4.1 and aquaporin-4 expression disrupts potassium and water homeostasis and may contribute to retinal edema. Activated Müller cells also become an important source of inflammatory and permeability-promoting mediators. In the diabetic retina, they upregulate interleukin-1β, tumor necrosis factor-α, monocyte chemoattractant protein-1, and VEGF, while the expression of protective factors such as pigment epithelium-derived factor and glial cell-derived neurotrophic factor may be reduced (24, 27, 28). Thus, Müller cell dysfunction converts a normally supportive glial interface into a source of inflammatory, angiogenic, and permeability-promoting signals.

Microglia, the resident immune cells of the retina, also become activated early in diabetes. They shift from a ramified homeostatic morphology toward reactive states characterized by cell body enlargement, process retraction, migration toward injured retinal layers, and accumulation around neurons and vessels (29). Reactive microglia release cytokines, chemokines, reactive oxygen species, and nitric oxide, which can amplify neuronal injury and vascular dysfunction (30). Rather than a simple M1/M2 dichotomy, diabetic microglial activation should be viewed as a spectrum of inflammatory and maladaptive states that contribute to chronic low-grade retinal inflammation. Astrocytes, which are mainly located in the nerve fiber layer and around retinal vessels, also undergo morphological and functional changes in DR. Their activation may alter the interaction between glial endfeet and endothelial cells, thereby impairing vascular tone regulation and BRB integrity (31). Collectively, glial dysfunction disrupts the communication bridge between neuronal activity and vascular response, making it a central driver of NVU uncoupling.

### 3.3 Vascular cell injury and blood–retinal barrier breakdown

Vascular cell injury remains a defining feature of DR and represents the vascular effector arm of NVU dysfunction. Retinal endothelial cells form the inner BRB through highly organized tight junctions, including claudin-5, occludin, and ZO-1. Under diabetic conditions, endothelial cells are exposed to hyperglycemia, oxidative stress, inflammatory cytokines, and altered growth factor signaling, all of which disrupt tight junction organization and increase vascular permeability (32, 33).

Endothelial dysfunction is accompanied by impaired nitric oxide bioavailability, increased leukocyte adhesion, and enhanced expression of adhesion molecules. These changes promote capillary occlusion, local hypoxia, and inflammatory cell recruitment (34, 35). Endothelial apoptosis further contributes to capillary dropout and nonperfusion, thereby creating hypoxic retinal areas that stimulate VEGF production and promote disease progression (36–38). Pericyte loss is another early and characteristic pathological feature of DR (16). Pericytes normally regulate capillary stability, endothelial survival, vascular tone, and BRB integrity through direct contact and paracrine signaling with endothelial cells (17). In diabetes, pericyte dropout weakens endothelial support, promotes microaneurysm formation, increases vascular permeability, and impairs autoregulatory responses (18). Loss of pericyte coverage also increases endothelial vulnerability to inflammatory and angiogenic stimuli.

The combined injury of endothelial cells and pericytes disrupts the vascular response required for neurovascular coupling. Even when neuronal or glial signals are generated, damaged capillaries may fail to produce an appropriate perfusion response. Therefore, vascular cell injury not only causes BRB breakdown and leakage but also impairs the vascular effector component of NVU coupling, predisposing the retina to edema, capillary nonperfusion, and progressive ischemia.

### 3.4 Extracellular matrix remodeling

**Figure 2:**
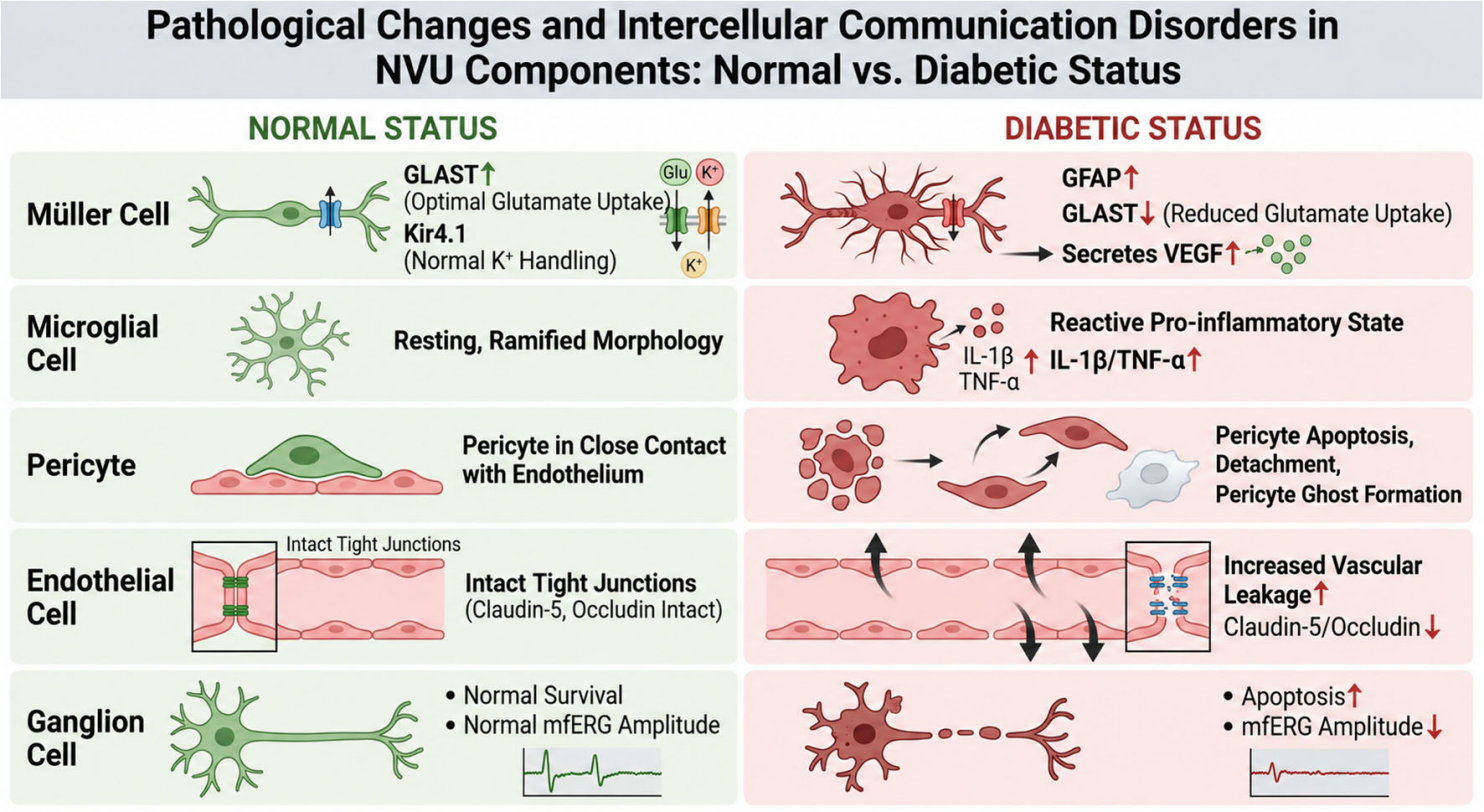
Pathological changes and intercellular communication disorders in neurovascular unit (NVU) components under diabetic stress. Chronic hyperglycemia induces distinct but interconnected changes in neuronal, glial, and vascular components of the NVU, including neuronal dysfunction and apoptosis, glial activation and neuroinflammation, endothelial injury, pericyte loss, and blood-retinal barrier breakdown. These changes collectively contribute to neurovascular uncoupling and diabetic retinopathy (DR) progression.

The extracellular matrix (ECM) provides structural support for the NVU and regulates cell adhesion, survival, migration, and signaling (39, 40). In the diabetic retina, ECM remodeling is characterized by basement membrane thickening, abnormal matrix accumulation, and altered matrix degradation. Excess deposition of type IV collagen, laminin, and fibronectin contributes to thickening of the capillary basement membrane, a classic histopathological feature of DR (41). Basement membrane thickening can impair endothelial-pericyte communication, reduce oxygen and nutrient diffusion, and facilitate pericyte detachment (13). At the same time, imbalance between matrix metalloproteinases and tissue inhibitors of metalloproteinases promotes abnormal matrix degradation, inflammatory cell infiltration, and BRB disruption (40). Upregulation of matrix metalloproteinase-2 (MMP-2) and matrix metalloproteinase-9 (MMP-9) has been implicated in basement membrane injury, vascular leakage, and proangiogenic remodeling (42). ECM remodeling therefore alters the physical and biochemical environment in which NVU cells interact. By weakening endothelial-pericyte adhesion, disturbing glial-vascular interfaces, and promoting inflammatory and angiogenic signaling, pathological ECM remodeling further destabilizes neurovascular communication and contributes to sustained NVU uncoupling. The major cell-specific alterations of the retinal NVU in DR are summarized in **Table 1** and illustrated in **Figure 2**.

**Table 1:** Cell-specific alterations of the neurovascular unit in diabetic retinopathy.

NVU component	Main diabetes-induced alterations	Key molecular or cellular mechanisms	Consequences for NVU uncoupling	Clinical or translational relevance
Photoreceptors and inner retinal neurons	Impaired synaptic transmission, altered glutamate signaling, reduced synaptic protein expression, and early functional abnormalities	Mitochondrial dysfunction, oxidative stress, glutamate excitotoxicity, impaired energy metabolism, and reduced neurotrophic support	Weakens activity-dependent vascular signaling and reduces the physiological trigger for neurovascular coupling	May explain early visual dysfunction before clinically visible vascular lesions; detectable by ERG/mfERG and retinal layer analysis
Retinal ganglion cells	Early apoptosis, reduced ganglion cell complex thickness, impaired electrophysiologic responses	Mitochondrial injury, caspase activation, endoplasmic reticulum stress, oxidative stress, and inflammatory cytokine exposure	Disrupts inner retinal signaling and contributes to early neurodegeneration-driven NVU dysfunction	Ganglion cell layer or ganglion cell complex thinning may serve as an early structural biomarker of diabetic retinal neurodegeneration
Muller glial cells	Reactive gliosis, increased GFAP expression, impaired glutamate clearance, abnormal Kir4.1 and aquaporin-4 expression, increased VEGF and inflammatory mediator production	Reduced GLAST/GLT-1 function, potassium and water homeostasis disruption, NF-kappaB/STAT3 activation, VEGF upregulation, and reduced PEDF/GDNF support	Converts a normally supportive glial interface into a source of inflammatory, angiogenic, and permeability-promoting signals	Central target for therapies aimed at restoring glial homeostasis, reducing edema, and protecting neurons
Microglia	Early activation, morphological transformation, migration toward injured neurons and vessels, and persistent low-grade inflammation	Activation by hyperglycemia, AGEs, ROS, DAMPs, and NLRP3 inflammasome signaling; release of IL-1beta, TNF-alpha, IL-6, nitric oxide, and ROS	Amplifies neuronal injury, endothelial dysfunction, and glial maladaptation, thereby sustaining inflammatory NVU uncoupling	Microglial modulation and inflammasome inhibition represent potential anti-inflammatory strategies for early DR
Astrocytes	Reactive gliosis, increased GFAP expression, vascular-associated remodeling, and altered interaction with endothelial cells	Inflammatory activation, oxidative stress, altered glial endfeet signaling, and impaired support of vascular tone regulation	Disturbs the glial-vascular interface and impairs local vascular autoregulation	May contribute to early BRB dysfunction and abnormal retinal perfusion regulation
Retinal endothelial cells / inner BRB interface	Tight junction disruption, increased permeability, endothelial apoptosis, leukocyte adhesion, and capillary nonperfusion	VEGF overexpression, oxidative stress, PKC activation, AGE-RAGE signaling, NF-kappaB activation, reduced NO bioavailability, and ICAM-1/VCAM-1 upregulation	Impairs the vascular effector arm of neurovascular coupling and promotes BRB breakdown, leakage, ischemia, and edema	Major target of anti-VEGF therapy, vascular-stabilizing therapy, and anti-inflammatory treatment
Pericytes	Pericyte dropout, reduced endothelial support, impaired capillary tone regulation, and microaneurysm formation	Oxidative stress, AGE-RAGE activation, impaired PDGF-B/PDGFR-beta signaling, reduced Ang-1/Tie-2 signaling, and apoptosis	Loss of pericyte support destabilizes capillaries, weakens autoregulation, and predisposes the retina to leakage and nonperfusion	Early pericyte loss is a hallmark of DR and supports therapeutic interest in vascular stabilization and Ang/Tie pathway modulation
Retinal pigment epithelial cells	Impaired outer BRB function, altered VEGF/PEDF balance, and contribution to inflammatory and angiogenic signaling	Hyperglycemia-induced oxidative stress, mitochondrial dysfunction, VEGF upregulation, PEDF reduction, and inflammatory mediator release	Disrupts outer retinal homeostasis and may aggravate vascular leakage, edema, and photoreceptor stress	Relevant to BRB integrity and may influence the response to antiangiogenic and barrier-stabilizing therapies
Extracellular matrix and basement membrane	Basement membrane thickening, abnormal accumulation of collagen IV, laminin, and fibronectin, and altered ECM degradation	Hyperglycemia-induced ECM synthesis, glycation-related matrix cross-linking, MMP-2/MMP-9 activation, and TIMP imbalance	Weakens endothelial-pericyte adhesion, disrupts glial-vascular communication, and promotes vascular leakage and inflammatory infiltration	ECM remodeling may contribute to irreversible microvascular dysfunction and represents a potential target for anti-fibrotic or matrix-modulating strategies

AGE, advanced glycation end product; BRB, blood–retinal barrier; DAMPs, damage-associated molecular patterns; DR, diabetic retinopathy; ECM, extracellular matrix; ERG, electroretinography; GFAP, glial fibrillary acidic protein; GDNF, glial cell-derived neurotrophic factor; ICAM-1, intercellular adhesion molecule-1; IL, interleukin; MMP, matrix metalloproteinase; mfERG, multifocal electroretinography; NO, nitric oxide; NVU, neurovascular unit; PEDF, pigment epithelium-derived factor; PKC, protein kinase C; ROS, reactive oxygen species; TIMP, tissue inhibitor of metalloproteinase; TNF-alpha, tumor necrosis factor-alpha; VCAM-1, vascular cell adhesion molecule-1; VEGF, vascular endothelial growth factor.

## 4 Molecular mechanisms of neurovascular unit uncoupling

**Figure 3:**
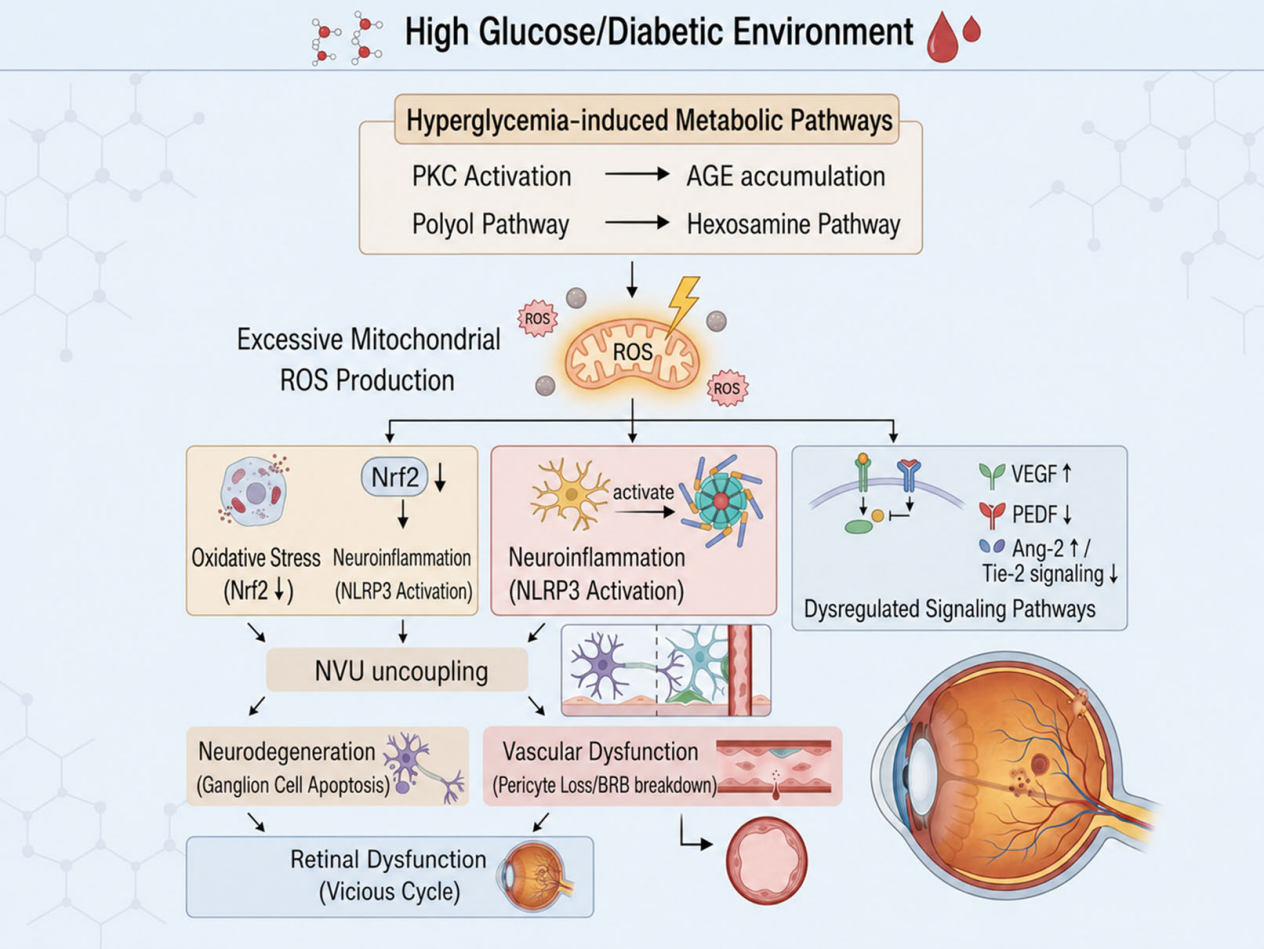
Molecular mechanisms driving neurovascular unit uncoupling in diabetic retinopathy. Hyperglycemia activates major metabolic injury pathways and increases mitochondrial reactive oxygen species (ROS) production. Oxidative stress, neuroinflammation, and dysregulated vascular endothelial growth factor (VEGF)/pigment epithelium-derived factor (PEDF) and angiopoietin-Tie (Ang/Tie) signaling further disrupt neurovascular homeostasis, creating a vicious cycle in which neurodegeneration and microvascular damage exacerbate each other.

NVU uncoupling in DR is not caused by a single pathway. Chronic hyperglycemia initiates a network of mutually reinforcing mechanisms, including mitochondrial reactive oxygen species (ROS) production, inflammatory activation, VEGF/angiopoietin-2 (Ang-2) imbalance, impaired neurotrophic support, disrupted intercellular communication, and epigenetic memory. These mechanisms reflect the local retinal effects of diabetes-induced metabolic stress and converge on three major outcomes: neuronal dysfunction, glial maladaptation, and vascular barrier failure. Once established, these processes amplify each other and drive the transition from early functional impairment to clinically visible retinopathy, diabetic macular edema, and proliferative disease. The major molecular pathways driving NVU uncoupling in DR are summarized in **Figure 3**.

### 4.1 Hyperglycemia-induced metabolic dysregulation and multicellular NVU injury

Hyperglycemia-induced metabolic dysregulation is the upstream trigger of NVU injury in DR (43). Excess intracellular glucose increases mitochondrial substrate flux and promotes ROS generation through the electron transport chain (44). This oxidative burden activates several interrelated pathogenic pathways, including the protein kinase C (PKC) pathway, advanced glycation end product (AGE) formation, the polyol pathway, and the hexosamine pathway (45, 46). Although these pathways are often described separately, they interact closely and ultimately converge on oxidative stress, inflammation, endothelial dysfunction, neuronal injury, and BRB breakdown.

PKC activation is an important mediator of hyperglycemic injury (47). Increased diacylglycerol synthesis activates several PKC isoforms, particularly PKC-β and PKC-δ. PKC activation increases VEGF expression, enhances vascular permeability, stimulates nicotinamide adenine dinucleotide phosphate (NADPH) oxidase-dependent ROS generation, activates nuclear factor-κB (NF-κB) signaling, and impairs eNOS function (45, 47). These effects simultaneously affect the neural, glial, and vascular components of the NVU.

The AGE–receptor for advanced glycation end products (RAGE) axis also contributes to chronic retinal injury (32). Binding of AGEs to RAGE on endothelial cells, pericytes, neurons, and glial cells activates NADPH oxidase, NF-κB, and mitogen-activated protein kinase (MAPK) signaling, leading to increased expression of inflammatory cytokines and adhesion molecules (48, 49). In pericytes, AGE–RAGE signaling promotes apoptosis and weakens PDGF-B/platelet-derived growth factor receptor-β (PDGFR-β)-mediated survival signaling (17). In neurons, AGEs induce oxidative stress and mitochondrial dysfunction, thereby contributing to apoptotic injury. The polyol pathway consumes nicotinamide adenine dinucleotide phosphate (NADPH) and reduces glutathione regeneration, thereby weakening antioxidant defense (45). The hexosamine pathway alters protein function through abnormal O-GlcNAcylation of key signaling proteins, including eNOS and protein kinase B (Akt) (46). Together, these metabolic abnormalities create a persistent injurious environment that impairs neuronal activity, glial homeostasis, and vascular stability, thereby initiating NVU uncoupling. This upstream role of hyperglycemia-induced metabolic stress indicates that early DR is not a purely vascular disorder, but a multicellular NVU injury process. It also supports the rationale for prioritizing systemic metabolic control and metabolic pathway modulation before irreversible neuronal, glial, and vascular damage becomes established.

### 4.2 Oxidative stress and impaired antioxidant defense

Oxidative stress is a central mechanism linking metabolic dysfunction to structural and functional NVU injury (50). In the diabetic retina, ROS are generated from multiple sources, including the mitochondrial electron transport chain, NADPH oxidase, xanthine oxidase, uncoupled nitric oxide synthase, and cytochrome P450 enzymes (16, 51). The overproduction of ROS, combined with insufficient antioxidant defense, produces a sustained redox imbalance that affects all NVU components.

Mitochondria are a major source of hyperglycemia-induced ROS (17, 44). Excess glucose increases mitochondrial substrate supply and enhances electron leakage at complexes I and III, resulting in superoxide generation. Mitochondrial ROS directly damage mitochondrial DNA, proteins, and lipids, but they also act as signaling molecules that activate PKC, MAPK, and NF-κB pathways (52, 53). In retinal neurons, mitochondrial dysfunction contributes to impaired energy metabolism and apoptosis. In glial cells, ROS promote reactive gliosis and inflammatory mediator release. In endothelial cells and pericytes, ROS disrupt tight junction integrity, impair survival signaling, and increase vascular permeability.

NADPH oxidase (NOX) further amplifies oxidative injury. NOX isoforms, including NOX1, NOX2, and NOX4, are expressed in retinal cells and can be activated by PKC, AGEs, angiotensin II, and inflammatory cytokines (47, 54). NOX2 is closely associated with inflammatory cells and activated microglia, whereas NOX4 contributes to endothelial dysfunction and BRB disruption (10, 30). This crosstalk between oxidative stress and inflammation is a key mechanism by which local retinal injury becomes self-sustaining. Antioxidant defense is also impaired in DR. The expression or activity of superoxide dismutase, catalase, and glutathione peroxidase may be reduced in the diabetic retina (54, 55). Nuclear factor erythroid 2-related factor 2 (Nrf2), a major transcriptional regulator of antioxidant responses, is suppressed under diabetic conditions, leading to insufficient expression of downstream antioxidant and detoxifying enzymes (55). As a result, retinal cells become less able to counteract chronic oxidative stress. Nitrosative stress provides another layer of injury. Superoxide reacts rapidly with nitric oxide to form peroxynitrite, which induces protein nitration and impairs the function of antioxidant enzymes, signaling proteins, and structural proteins (45, 56). Peroxynitrite formation also reduces nitric oxide bioavailability, thereby weakening the vasodilatory responses required for neurovascular coupling. In this context, oxidative and nitrosative stress act not only as direct sources of retinal cell injury but also as amplifiers of inflammation, vascular leakage, and impaired blood-flow regulation. This provides a mechanistic basis for considering antioxidant and mitochondrial-protective strategies as early adjunctive approaches within a stage-adapted NVU-oriented treatment framework.

### 4.3 Initiation and persistence of neuroinflammation

Neuroinflammation is a major mechanism through which diabetic stress is translated into progressive NVU dysfunction (23, 34). It involves reactive glial changes, cytokine and chemokine release, leukocyte adhesion, complement activation, and immune cell recruitment. This chronic low-grade inflammatory state promotes both neurodegeneration and vascular injury, thereby accelerating DR progression (35).

Microglia play a central role in initiating and maintaining retinal inflammation (29, 30). In early diabetes, hyperglycemia, AGEs, oxidative stress, and damage-associated molecular patterns activate microglia and shift them toward reactive pro-inflammatory states. These reactive microglia express inflammatory mediators such as inducible nitric oxide synthase (iNOS) and cyclooxygenase-2 (COX-2) and release interleukin-1β (IL-1β), tumor necrosis factor-α (TNF-α), interleukin-6 (IL-6), nitric oxide, and ROS, thereby exerting toxic effects on neurons and vascular cells. Rather than following a simple M1/M2 dichotomy, diabetic microglia may adopt a spectrum of reactive states characterized by inflammatory cytokine release, oxidative stress, altered phagocytosis, and impaired homeostatic support. The NOD-like receptor protein 3 (NLRP3) inflammasome is an important molecular platform for inflammatory activation in DR (34). In retinal microglia and Müller cells, hyperglycemia, ROS, and AGEs can activate NLRP3, leading to caspase-1 activation and maturation of IL-1β and interleukin-18 (IL-18). These cytokines amplify local inflammation, promote apoptosis, and increase vascular permeability. Inflammasome activation therefore links metabolic stress with both neuronal injury and BRB disruption.

Pro-inflammatory cytokines injure the NVU through multiple mechanisms (35, 57). Tumor necrosis factor-α (TNF-α) activates nuclear factor-κB (NF-κB) and c-Jun N-terminal kinase (JNK) signaling, increases intercellular adhesion molecule-1 (ICAM-1) and vascular cell adhesion molecule-1 (VCAM-1) expression, promotes leukocyte adhesion, induces endothelial apoptosis, and increases vascular leakage (34, 35). IL-1β amplifies inflammatory signaling, stimulates additional cytokines and chemokines, activates MMP-9, and promotes matrix degradation and edema formation (39, 40). IL-6 activates Janus kinase (JAK)/signal transducer and activator of transcription 3 (STAT3) signaling and has been associated with inflammation, angiogenesis, and DR severity. Leukostasis is another early inflammatory vascular event in DR. Adherent leukocytes release ROS and proteases, obstruct capillaries, injure endothelial cells, and disrupt the BRB during transmigration (34). Complement activation may further aggravate inflammation, as complement components and membrane attack complex deposition have been reported in diabetic retinal tissues (58). The combined effects of glial inflammation, leukocyte-mediated endothelial injury, and complement activation establish a feed-forward inflammatory loop that converts transient metabolic stress into persistent NVU damage and progressive neurovascular uncoupling. In this sense, neuroinflammation functions as a key transition mechanism between early metabolic injury and sustained structural and functional NVU failure. This provides a rationale for anti-inflammatory and glial-modulating strategies, particularly in patients or disease stages characterized by inflammatory activation, BRB disruption, or an incomplete response to anti-permeability therapy alone.

### 4.4 Disrupted VEGF/Ang-Tie and neurotrophic signaling

Precise intercellular signaling is essential for maintaining NVU coupling. In the healthy retina, neuronal activity is translated into vascular responses through coordinated signals from neurons, Müller cells, astrocytes, endothelial cells, and pericytes. Diabetes disrupts these signaling networks, resulting in impaired vascular stability, abnormal permeability, and loss of activity-dependent blood flow regulation.

The VEGF pathway is the most extensively studied signaling axis in DR (59, 60). Hypoxia, oxidative stress, AGEs, and inflammatory cytokines increase VEGF production, mainly from Müller cells, retinal ganglion cells, and the retinal pigment epithelium (24, 27). Excessive VEGF activates VEGF receptor 2 (VEGFR2)-mediated phospholipase Cγ (PLCγ)/PKC, phosphoinositide 3-kinase (PI3K)/Akt, and MAPK signaling in endothelial cells, leading to tight junction phosphorylation, endothelial permeability, and angiogenic activation (40). Although VEGF has physiological roles in neuronal and vascular survival, sustained VEGF overexpression in diabetes shifts the balance toward leakage, edema, and neovascularization.

The Ang/Tie pathway is another key regulator of vascular stability (61). Ang-1 normally activates Tie-2 signaling and supports endothelial survival, pericyte interaction, and barrier integrity. In the diabetic retina, Ang-1 signaling is reduced, whereas Ang-2 is increased, weakening Tie-2-mediated vascular stabilization (62). This imbalance promotes pericyte dropout, vascular destabilization, inflammation, and increased permeability. The clinical efficacy of dual VEGF-A/Ang-2 inhibition in diabetic macular edema supports the relevance of this pathway as a therapeutic target.

PDGF-B/PDGFR-β signaling is essential for pericyte recruitment and survival (17, 18). Impairment of this pathway reduces pericyte coverage and weakens endothelial-pericyte communication. As pericytes are important vascular effectors of neurovascular coupling, disruption of PDGF signaling contributes not only to capillary instability but also to impaired autoregulatory responses. Neurotrophic signaling is also reduced in DR. Decreased expression of brain-derived neurotrophic factor, glial cell-derived neurotrophic factor, nerve growth factor, and pigment epithelium-derived factor weakens neuronal survival and glial support (13, 27). Reduced pigment epithelium-derived factor also disturbs the VEGF/PEDF balance, favoring angiogenesis and permeability. Together, impaired angiogenic balance, vascular stabilization signaling, and neurotrophic support provide a molecular basis for the simultaneous development of neuronal injury and vascular leakage in DR. This disrupted signaling network provides a mechanistic bridge between NVU uncoupling and clinically relevant outcomes, including vascular leakage, diabetic macular edema, and neovascular progression. It also supports stage-adapted vascular stabilization strategies, including VEGF blockade, Ang-2/Tie-2 modulation, and combination approaches that address both permeability and endothelial-pericyte instability.

### 4.5 Epigenetic modifications and metabolic memory

Epigenetic modification provides a molecular explanation for metabolic memory in DR, in which early hyperglycemic exposure produces persistent pathogenic effects even after glycemic control improves (55, 63). This phenomenon highlights why early intervention is critical and why retinal injury may continue despite later systemic metabolic correction. DNA methylation patterns are altered in the diabetic retina. Hypomethylation of promoters of pro-inflammatory genes may sustain cytokine expression, whereas hypermethylation of protective genes, including Nrf2 and sirtuin 1 (SIRT1), may suppress antioxidant and cytoprotective pathways (63). Histone modifications also contribute to persistent gene dysregulation. Increased activating marks, such as H3K9ac and H3K4me3, at inflammatory gene promoters and reduced repressive marks, such as H3K9me3, can maintain the overexpression of pathogenic genes (48, 55).

Noncoding RNAs are another important layer of epigenetic regulation. Several microRNAs (miRNAs) are dysregulated in DR. miR-200b regulates VEGF expression and angiogenesis, miR-146a modulates inflammatory signaling, miR-195 affects oxidative stress through SIRT1-related pathways, and miR-126 supports endothelial integrity (64, 65). Long noncoding RNAs, including MALAT1, ANRIL, and MIAT, participate in inflammation, angiogenesis, and endothelial dysfunction through miRNA sponging and transcriptional regulation (65). These epigenetic mechanisms may establish a stable pathogenic transcriptional program involving oxidative stress, inflammation, endothelial dysfunction, and neuronal vulnerability. By maintaining abnormal gene expression after the initial metabolic insult, epigenetic memory may help explain why NVU uncoupling can persist and progress even when systemic risk factors are subsequently improved. This persistence reinforces the importance of treatment timing in DR. Once epigenetically maintained injury programs are established, later vascular-directed therapy may control edema, leakage, or neovascular complications but may not fully reverse earlier neuronal, glial, and microvascular damage. These observations support earlier and mechanism-based intervention within a stage-adapted NVU-oriented therapeutic framework.

### 4.6 Abnormal intercellular communication

Functional coupling within the NVU depends on direct cell contact, paracrine signaling, gap junction communication, and extracellular vesicle-mediated molecular exchange. Diabetes disrupts each of these communication routes, thereby weakening the coordinated response of the retina to metabolic demand. Gap junctions formed by connexins allow the transfer of ions, metabolites, and second messengers between adjacent cells. Connexin 43 is expressed in astrocytes, Müller cells, and endothelial cells, whereas connexin 40 and connexin 37 are mainly associated with endothelial communication (13). In diabetes, connexin expression, phosphorylation, and membrane localization may be altered, impairing glial network communication, potassium buffering, and metabolite clearance. Dysfunction of endothelial gap junctions may also reduce the propagation of vasodilatory signals along the microvasculature.

Endothelial–pericyte communication is also impaired. Under physiological conditions, pericytes maintain endothelial stability through direct peg-socket contacts, gap junctions, Ang-1/Tie-2 signaling, and TGF-β-related pathways, whereas endothelial cells provide PDGF-B to support pericyte recruitment and survival (16, 17). Diabetes weakens these reciprocal signals, reduces pericyte coverage, and disrupts endothelial-pericyte contact (18). This loss of vascular cellular communication directly compromises capillary stability and perfusion regulation. Extracellular vesicles, including exosomes and microvesicles, are emerging mediators of intercellular communication in DR. They transfer proteins, lipids, and nucleic acids, particularly miRNAs, between retinal cells. In the diabetic retina, extracellular vesicles may carry pro-inflammatory, pro-apoptotic, or proangiogenic cargo. For example, endothelial cell-derived vesicles with altered miRNA content may affect recipient glial or vascular cells, while vesicles released from activated inflammatory cells may amplify local injury (65). Changes in circulating or intraocular extracellular vesicles may also serve as biomarkers of DR activity. More broadly, disrupted intercellular communication provides a final common pathway linking cellular injury and molecular dysregulation. When neuronal, glial, endothelial, and pericyte signals are no longer synchronized, the retina loses its ability to coordinate neural activity, vascular perfusion, barrier integrity, and immune homeostasis. This breakdown of communication is therefore central to sustained NVU uncoupling in DR and suggests that therapeutic strategies should not only suppress individual pathogenic pathways, but also preserve or restore coordinated neuronal, glial, endothelial, and pericyte interactions across different stages of disease.

## 5 NVU-oriented therapeutic targets and stage-adapted strategies

Conventional treatments for DR mainly target mid- to late-stage vascular complications, including panretinal photocoagulation (PRP), intravitreal anti-vascular endothelial growth factor (anti-VEGF) therapy, and vitrectomy. Although these approaches can control proliferative disease and diabetic macular edema (DME), they do not reverse neurodegeneration or early NVU injury (23). This limitation highlights the need to view DR therapy through a broader NVU-based and stage-dependent framework. Because DR is biologically heterogeneous, the dominant pathogenic process may differ across patients and disease stages, including metabolic stress, oxidative injury, neuroinflammation, glial dysfunction, vascular leakage, ischemia, and proliferative remodeling. Therefore, NVU-oriented treatment should not be interpreted as a single therapeutic pathway, but as a mechanism-based framework that matches therapeutic targets to the dominant biological process and translational maturity of each intervention.

**Figure 4:**
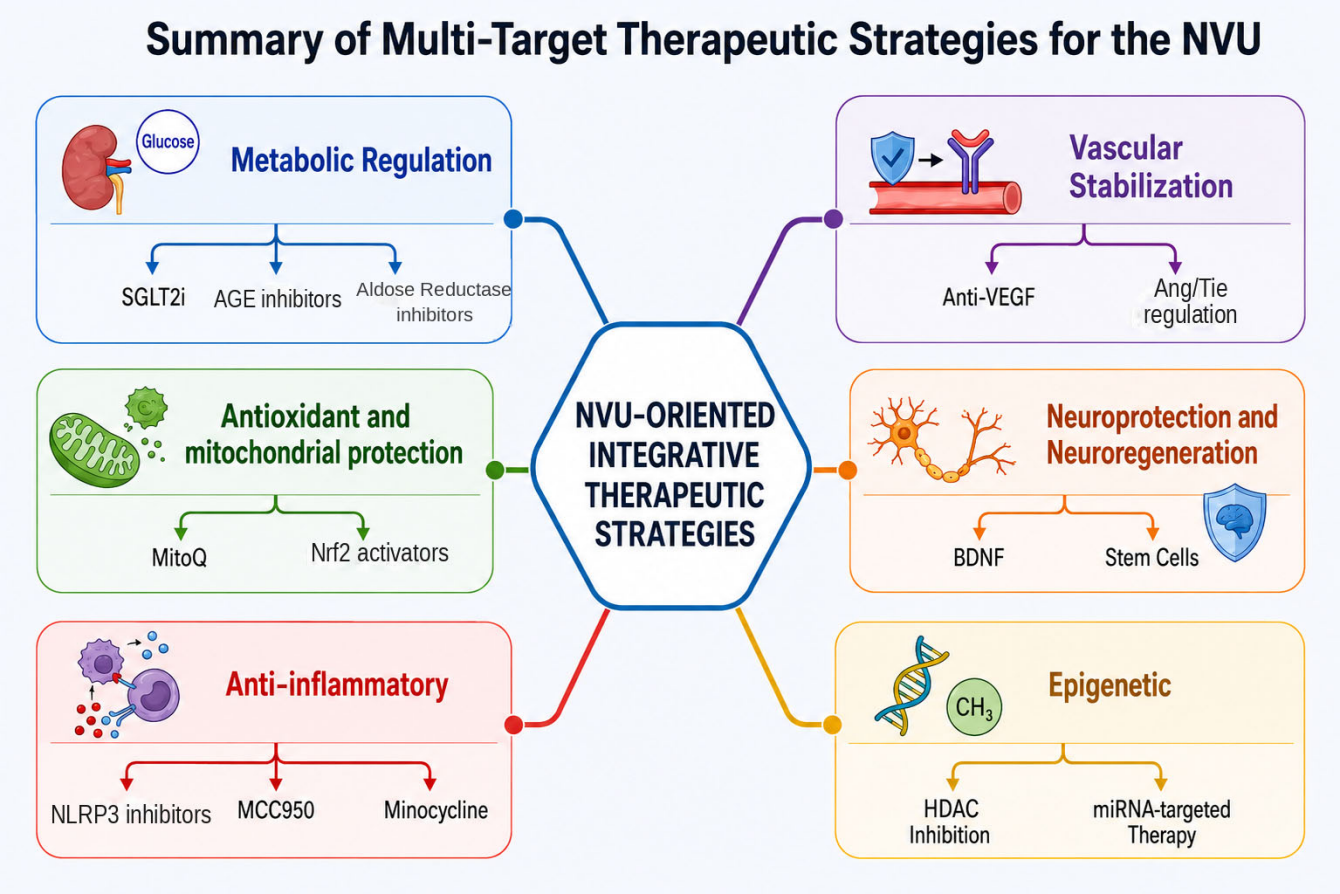
Multi-target therapeutic strategies for restoring neurovascular unit coupling in diabetic retinopathy. Therapeutic approaches targeting neurovascular unit (NVU) dysfunction include metabolic regulation, antioxidant and mitochondrial protection, anti-inflammatory therapy, neuroprotection and neuroregeneration, vascular stabilization, and epigenetic modulation. These integrated strategies may help preserve retinal homeostasis and slow disease progression.

With a better understanding of DR pathogenesis, multimodal strategies targeting the NVU and applied at an earlier stage have become an active area of investigation (41, 66). For clinical translation, NVU-targeted therapies can be broadly classified into three categories: clinically established vascular therapies, therapies with early clinical or indirect systemic evidence, and preclinical approaches requiring further validation. Representative NVU-oriented therapeutic strategies and their translational status are summarized in **Figure 4** and **Table 2**.

**Table 2:** Therapeutic strategies targeting retinal NVU dysfunction and their translational status in diabetic retinopathy.

Therapeutic strategy	Main NVU target or pathway	Representative approaches	Evidence level	Clinical or translational status
Glycemic and metabolic control	Upstream metabolic stress, metabolic memory, systemic vascular risk	Intensive glycemic control, blood pressure and lipid control, metformin, GLP-1 receptor agonists, SGLT2 inhibitors	Large clinical trials for systemic risk control; indirect retinal evidence for newer agents	Cornerstone of DR prevention and systemic management
AGE-RAGE and polyol pathway targeting	AGE accumulation, RAGE-mediated inflammation, sorbitol accumulation, oxidative stress	AGE formation inhibitors, AGE breakers, RAGE antagonists, soluble RAGE, aldose reductase inhibitors	Mainly preclinical; limited clinical translation	Investigational retinal NVU-protective strategy
Antioxidant and mitochondrial protection	Mitochondrial ROS production, NADPH oxidase activation, impaired antioxidant defense	MitoQ, MitoTEMPO, Alda-1, NOX inhibitors, SOD mimetics, Nrf2 activators, resveratrol, curcumin	Mainly preclinical	Investigational; potential early-stage NVU-protective approach
Anti-VEGF therapy	VEGF-mediated vascular leakage, macular edema, and neovascularization	Ranibizumab, aflibercept, bevacizumab, brolucizumab	Large randomized clinical trials and real-world studies	Standard therapy for DME and proliferative DR
Dual VEGF-A/Ang-2 inhibition and Tie-2 pathway modulation	VEGF-A-driven leakage, Ang-2-mediated Tie-2 destabilization, vascular instability	Faricimab; Ang/Tie pathway modulation	Phase 3 randomized clinical trials	Approved and clinically validated for DME; represents vascular stabilization beyond VEGF blockade alone
Corticosteroids and broad anti-inflammatory therapy	Retinal inflammation, BRB leakage, VEGF expression, Muller cell and microglial activation	Dexamethasone implant, fluocinolone acetonide implant, intravitreal triamcinolone	Clinical trials and real-world studies	Established therapy for DME, especially in pseudophakic eyes or in patients with insufficient response to anti-VEGF therapy
Targeted immunomodulation and inflammasome inhibition	Cytokine signaling, NLRP3 inflammasome activation, complement activation, leukostasis	Anti-TNF-alpha agents, IL-1 pathway inhibitors, NLRP3 inhibitors, complement inhibitors	Mainly preclinical; small or indirect clinical evidence	Investigational anti-inflammatory NVU-targeted strategy
Microglial and glial modulation	Sustained microglial activation, Muller cell dysfunction, glutamate imbalance, impaired ion and water homeostasis	Minocycline, PPAR-gamma modulation, TREM2-related approaches, GLT-1 upregulation, Kir4.1/AQP4 modulation, Cx43-targeted approaches	Mainly preclinical or early translational evidence	Investigational; potentially useful for early inflammatory or neurodegenerative phenotypes
Neurotrophic and neuroprotective therapy	Retinal neuronal survival, synaptic function, neurotrophic support, ganglion cell injury	BDNF, NGF, GDNF, PEDF, citicoline, EPO analogues, retinoic acid receptor agonists, tacrolimus	Preclinical evidence; limited small clinical studies for selected agents	Potential adjunctive strategy for early neurodegenerative DR
Epigenetic and RNA-based therapy	Metabolic memory, persistent inflammatory transcription, angiogenic imbalance, endothelial dysfunction	HDAC inhibitors, DNA methylation modulators, miR-200b mimics, miR-126 supplementation, antagomiRs, lncRNA-targeted therapy	Mainly preclinical	Investigational; may target persistent disease programming
Gene therapy and sustained-release delivery systems	Long-term intraocular expression or sustained delivery of antiangiogenic, anti-inflammatory, or neuroprotective molecules	AAV-mediated gene delivery, nonviral vectors, nanoparticles, sustained-release implants, port delivery systems	Established for some delivery platforms; investigational for most NVU-specific targets	Enabling platform for reducing treatment burden and improving posterior segment delivery
Stem cell and cell-based therapy	Neurovascular repair, paracrine trophic support, inflammation modulation, vascular stabilization	Mesenchymal stem cells, induced pluripotent stem cell-derived retinal cells, retinal organoid-derived approaches	Preclinical and early clinical studies	Experimental regenerative strategy

AAV, adeno-associated virus; AGE, advanced glycation end product; AQP4, aquaporin-4; BRB, blood–retinal barrier; Cx43, connexin 43; DME, diabetic macular edema; DR, diabetic retinopathy; EPO, erythropoietin; GDNF, glial cell-derived neurotrophic factor; GLP-1, glucagon-like peptide-1; GLT-1, glutamate transporter-1; HDAC, histone deacetylase; IL, interleukin; lncRNA, long non-coding RNA; NGF, nerve growth factor; NOX, NADPH oxidase; NVU, neurovascular unit; PEDF, pigment epithelium-derived factor; RAGE, receptor for advanced glycation end products; ROS, reactive oxygen species; SGLT2, sodium–glucose cotransporter 2; SOD, superoxide dismutase; TNF-alpha, tumor necrosis factor-alpha; VEGF, vascular endothelial growth factor.

### 5.1 Metabolic regulation and advanced glycation end product targeting

Strict glycemic control remains the foundation of DR prevention and treatment. Large clinical trials, including the Diabetes Control and Complications Trial/Epidemiology of Diabetes Interventions and Complications (DCCT/EDIC) and the United Kingdom Prospective Diabetes Study (UKPDS), have shown that intensive glucose control reduces the risk of DR and slows its progression (67). However, the benefit of glycemic control is time-dependent, and early intervention provides more durable protection, consistent with metabolic memory (55, 56). Newer glucose-lowering agents, including glucagon-like peptide-1 (GLP-1) receptor agonists and sodium-glucose cotransporter 2 (SGLT2) inhibitors, may improve systemic metabolic and vascular risk factors relevant to DR. However, their direct retinal effects remain incompletely defined. The association between GLP-1 receptor agonists and DR progression may depend on baseline retinopathy severity, the speed of glycemic improvement, and systemic risk profiles. Therefore, these agents should be discussed as systemic modifiers of diabetes-related retinal risk rather than established retinal NVU-targeted therapies. Several approaches target the AGE–RAGE axis (32). AGE formation inhibitors, such as aminoguanidine, block AGE generation and attenuate retinal vascular injury and neural damage in animal models, although clinical use has been limited by toxicity. AGE breakers, such as N-phenylthiazolium bromide, cleave preformed AGE cross-links and reduce AGE accumulation in models of diabetic complications. RAGE antagonists inhibit downstream inflammatory and oxidative signaling, while soluble RAGE acts as a decoy receptor that binds AGEs and prevents interaction with cell-surface RAGE. Both approaches have shown protective effects in experimental DR. Aldose reductase inhibitors suppress the polyol pathway, reducing sorbitol accumulation and NADPH consumption (45). Several aldose reductase inhibitors have shown retinal protection in preclinical studies, including reduced neuronal apoptosis, improved electrophysiologic function, and decreased vascular leakage.

### 5.2 Oxidative stress targeted therapy

Mitochondria-targeted antioxidants are conjugated to lipophilic cations such as triphenylphosphonium. This allows selective accumulation within mitochondria and scavenging of ROS at a major site of production (68, 69). Agents such as MitoQ and MitoTEMPO have shown protective effects in animal models of DR, including reduced mitochondrial injury, inhibition of apoptosis, improved retinal function, and decreased vascular leakage. Alda-1, an activator of mitochondrial aldehyde dehydrogenase 2, reduces oxidative injury by clearing toxic aldehydes and has shown benefit in diabetic cardiovascular complications. Its role in DR warrants further study (70). Nrf2 activators enhance endogenous antioxidant defense. Sulforaphane and tert-butylhydroquinone activate Nrf2 and upregulate antioxidant and detoxifying enzymes, including heme oxygenase-1 (HO-1), NAD(P)H quinone oxidoreductase 1 (NQO1), and superoxide dismutase (SOD) (55). In DR models, Nrf2 activation reduces oxidative stress, inflammation, and neurovascular injury. Natural compounds, such as resveratrol, curcumin, and anthocyanins, may also act through Nrf2-mediated antioxidant and anti-inflammatory effects. Bardoxolone methyl, a synthetic Nrf2 activator, improved renal function in trials of diabetic nephropathy, but cardiovascular safety concerns led to discontinuation. Its use in DR requires further evaluation. NOX inhibitors reduce ROS generation by blocking NADPH oxidase. Pan NOX inhibitors, such as apocynin and VAS2870, attenuate oxidative stress, inflammation, and vascular leakage in DR models. Isoform-selective inhibitors, such as the NOX1/4 inhibitor GKT137831, are being studied in cardiovascular and fibrotic diseases and may have relevance for DR (54). SOD mimetics, such as Tempol, scavenge superoxide and reduce oxidative injury, with reported neuroprotective and vasculoprotective effects in DR models.

### 5.3 Anti-inflammatory and immunomodulatory strategies

Nonsteroidal anti-inflammatory drugs (NSAIDs) reduce prostaglandin synthesis by inhibiting cyclooxygenase. The COX-2 selective inhibitor celecoxib reduced inflammation, leukocyte adhesion, and vascular leakage in DR models (34). Clinical studies suggest that topical NSAID eye drops, such as bromfenac and nepafenac, may be useful as adjunctive therapy for diabetic macular edema, although cardiovascular risk limits systemic NSAID use (35).

Glucocorticoids have potent anti-inflammatory activity. Intravitreal corticosteroids, including triamcinolone and dexamethasone implants, are used for diabetic macular edema and can reduce inflammation, suppress VEGF, and stabilize the BRB (41). However, elevated intraocular pressure and cataract formation limit long-term use. Glucocorticoid receptor agonists with fewer adverse effects remain an active area of research. Biologics targeting specific inflammatory mediators have also shown promise in DR (57). Anti-TNF-α agents, such as adalimumab and infliximab, are widely used in inflammatory diseases, and small studies in DR suggest potential benefit for macular edema. Larger clinical trials are still needed. The interleukin-1 (IL-1) receptor antagonist anakinra and the anti-interleukin-1β (anti-IL-1β) antibody canakinumab reduce inflammation by blocking IL-1 signaling and have shown benefit in trials of diabetic cardiovascular complications, supporting further study in DR (34, 35). NLRP3 inflammasome inhibitors, such as MCC950, selectively suppress NLRP3 activation, block caspase-1 activation and IL-1β maturation, and reduce inflammation, neural injury, and vascular leakage in DR models.

Microglial modulation is an important anti-inflammatory target (29). Minocycline, beyond its antimicrobial activity, suppresses microglial activation and reduces neuroinflammation and neuronal apoptosis in DR models (30). Strategies that reprogram microglia from sustained pro-inflammatory states toward reparative or homeostatic states may help limit chronic retinal inflammation and support tissue repair. Potential approaches include modulation of interleukin-4/interleukin-13 (IL-4/IL-13)-related signaling, activation of peroxisome proliferator-activated receptor-γ (PPAR-γ), and enhancement of triggering receptor expressed on myeloid cells 2 (TREM2)-mediated microglial homeostatic functions, although their therapeutic relevance in DR requires further validation. Complement inhibition is also being explored. C5 inhibitors, such as eculizumab and ravulizumab, are already used in complement-mediated diseases, and experience from trials in age-related macular degeneration may inform DR research. Factor D inhibitors and C3 inhibitors targeting the alternative pathway may provide broader complement suppression (58).

### 5.4 Neuroprotection and neuroregeneration

Neurotrophic factor supplementation is a direct neuroprotective strategy. Brain-derived neurotrophic factor (BDNF), nerve growth factor (NGF), and glial cell-derived neurotrophic factor (GDNF) have shown neuronal protection, reduced apoptosis, and improved electrophysiologic function in DR models (13). However, poor BRB penetration and a short half-life limit the clinical translation of protein-based therapies. Gene therapy-mediated expression, small-molecule receptor agonists, and mimetic peptides are under development (38, 41). Citicoline is a nucleotide precursor involved in phospholipid synthesis and neurotransmitter release and has neuroprotective properties (23). It has shown benefit in neurodegenerative disorders such as glaucoma and stroke. Small studies suggest that citicoline may improve retinal electrophysiologic function in patients with DR, but larger trials are needed. Erythropoietin (EPO) exerts neuroprotective, anti-apoptotic, and anti-inflammatory effects through EPO receptors expressed on neurons, glial cells, and endothelial cells. EPO analogues that do not increase hematocrit, such as carbamylated EPO, have shown protection in DR models while avoiding erythropoietic adverse effects (71, 72). Retinoic acid receptor agonists may protect neurons through transcriptional regulation. 9-cis-retinoic acid reduced neurodegeneration and vascular leakage in DR models, possibly through anti-inflammatory, antioxidant, and VEGF-modulating effects. Tacrolimus, a calcineurin inhibitor, protects neurons by suppressing calcium-dependent apoptotic signaling. Topical delivery avoids systemic immunosuppression and has shown neuroprotective effects in DR models. Stem cell therapy is a frontier in regenerative medicine (41). Mesenchymal stem cells exert multiple protective effects in DR models through paracrine release of neurotrophic, anti-inflammatory, and vasculoprotective factors, and potentially through differentiation into retinal cells. Reported benefits include reduced neurodegeneration, suppressed inflammation, vascular stabilization, and improved visual function. Induced pluripotent stem cell (iPSC)-derived retinal cells may provide a basis for cell replacement therapy. Clinical trials are now evaluating the safety and efficacy of stem cell therapy in DR.

### 5.5 Vascular stabilization and antiangiogenic therapy

Anti-VEGF therapy is now standard treatment for proliferative DR and diabetic macular edema (38). Bevacizumab, ranibizumab, aflibercept, and the newer agent brolucizumab neutralize VEGF, reduce vascular leakage and macular edema, and suppress neovascularization. Long-term studies, including Diabetic Retinopathy Clinical Research Network (DRCR) Protocol T and the RISE/RIDE trials, have confirmed substantial visual benefit. However, some patients respond poorly or develop tachyphylaxis, and repeated injections impose a significant treatment burden. Combination approaches, such as anti-VEGF therapy with corticosteroids or laser, may improve outcomes (73).

Modulation of the Ang/Tie pathway has emerged as an important therapeutic direction for diabetic macular edema. Faricimab, a bispecific antibody targeting VEGF-A and Ang-2, provides a clinically relevant example of combined anti-permeability and vascular-stabilizing therapy. By inhibiting VEGF-A, faricimab reduces VEGF-driven vascular leakage, while Ang-2 inhibition may help restore Tie-2-mediated endothelial stability and improve pericyte-endothelial interaction. In the YOSEMITE and RHINE trials, faricimab achieved non-inferior visual gains compared with aflibercept, together with robust anatomical improvement and extended treatment durability in many patients. These findings support Ang-2/Tie-2 modulation as a clinically important strategy that may complement VEGF blockade in DME (16, 41). Integrin antagonists block integrin-mediated cell-extracellular matrix (cell-ECM) and cell-cell interactions and thereby inhibit angiogenesis and inflammation (38). The αvβ3 integrin antagonist has been tested in neovascular eye disease, although efficacy has been limited. Because α5β1 integrin contributes to pathological angiogenesis, it remains a potential target.

Angiopoietin-like protein 4 (ANGPTL4) also regulates angiogenesis and metabolism. ANGPTL4 is upregulated in the diabetic retina and promotes vascular leakage, whereas anti-ANGPTL4 antibodies reduce macular edema in animal models. Notch signaling modulation may suppress pathological angiogenesis, but therapeutic targeting must preserve physiological vascular maintenance and avoid excessive vascular regression.

### 5.6 Restoration of intercellular signaling and glial modulation

Therapies targeting Müller cell dysfunction focus on restoring ion and neurotransmitter homeostasis. Activation of Kir4.1 channels may improve potassium buffering, but specific agents are lacking. Upregulation of glutamate transporters, for example through ceftriaxone-induced glutamate transporter-1 (GLT-1) transcription, may reduce excitotoxicity and provide neuroprotection (13). Modulating Müller cell inflammatory responses is another important strategy. Inhibition of nuclear factor-κB (NF-κB) or signal transducer and activator of transcription 3 (STAT3) signaling, or induction of a neuroprotective phenotype, has therapeutic potential (24, 49). Müller cells also retain some regenerative capacity, and activation of regenerative programs in mammalian Müller cells remains a frontier in regenerative medicine. PEDF supplementation can restore the VEGF/PEDF balance and provide antiangiogenic and neuroprotective effects. Recombinant PEDF protein, gene therapy, and small-molecule mimetics have shown protective effects in DR models (27, 38).

Pharmacologic modulation of neurovascular coupling is an emerging area. Improving nitric oxide bioavailability, for example by tetrahydrobiopterin (BH4) supplementation to restore eNOS coupling or by nitric oxide donors, may enhance vasodilation (45). Iloprost, a prostacyclin analogue, promotes vasodilation and anti-inflammatory signaling through prostacyclin receptor activation. Potassium channel openers improve blood flow regulation by hyperpolarizing vascular smooth muscle cells. In addition, gap junction modulators, such as connexin 43 (Cx43) mimetic peptides, have shown protection in ischemic retinal disease by regulating gap junction and hemichannel opening. Their role in DR is still under investigation.

### 5.7 Epigenetic therapy

Targeting epigenetic modifications to reverse abnormal gene expression programs is an emerging therapeutic strategy (55). Histone deacetylase (HDAC) inhibitors, such as valproic acid, trichostatin A (TSA), and suberoylanilide hydroxamic acid (SAHA), exert anti-inflammatory, antioxidant, and neuroprotective effects by increasing histone acetylation. However, lack of selectivity with pan-HDAC inhibitors raises safety concerns, and HDAC isoform-specific inhibitors, such as the HDAC6 inhibitor tubastatin A, may improve tolerability. DNA methylation inhibitors, such as 5-azacytidine, can reactivate protective genes, but systemic toxicity limits ophthalmic application, making safer local or milder modulation strategies necessary (63). Histone methylation modulators, such as lysine-specific demethylase 1 (LSD1) and enhancer of zeste homolog 2 (EZH2) inhibitors, may also be useful in regulating inflammatory and fibrotic gene expression. miRNA-based therapy includes antagomiRs and miRNA mimics (64, 65). Restoration of miR-200b expression using miR-200b mimics may suppress VEGF expression and reduce pathological vascular leakage and neovascularization, whereas miR-126 supplementation may improve endothelial integrity. Delivery efficiency, off-target effects, and stability remain major challenges, although locked nucleic acid (LNA) modification, phosphorothioate modification, and nanocarrier systems may improve therapeutic performance (65).

### 5.8 Metabolic reprogramming and mitochondrial function improvement

Improving mitochondrial function and metabolism may reduce NVU injury at its source. Activation of peroxisome proliferator-activated receptor-γ coactivator 1α (PGC-1α) promotes mitochondrial biogenesis, improves oxidative phosphorylation efficiency, and reduces ROS production (64, 68). Sirtuin activators, such as resveratrol and nicotinamide mononucleotide (NMN), improve mitochondrial function through the sirtuin 1 (SIRT1)/PGC-1α pathway and have shown benefit in DR models (74). Nicotinamide adenine dinucleotide (NAD+) precursors, including nicotinamide riboside and NMN, restore the ratio of NAD+ to reduced nicotinamide adenine dinucleotide (NADH) and support sirtuin activity, and their use in DR is being explored (48). AMP-activated protein kinase (AMPK) activators, such as metformin and 5-aminoimidazole-4-carboxamide ribonucleotide (AICAR), exert protective effects by improving energy metabolism and suppressing inflammation and oxidative stress. Epidemiologic studies suggest that metformin may reduce DR risk, at least in part independently of glucose lowering (67, 72). Regulation of mitochondrial dynamics is another important strategy. In diabetes, dynamin-related protein 1 (Drp1)-mediated fission is increased, whereas mitofusin 1 (Mfn1) and mitofusin 2 (Mfn2) expression is reduced (69). Inhibiting Drp1, for example with mitochondrial division inhibitor 1 (Mdivi-1), or promoting fusion may improve mitochondrial function. Enhancing mitophagy, for example through activation of the PTEN-induced kinase 1 (PINK1)-Parkin pathway, may help remove damaged mitochondria and maintain quality control. Ketone metabolism is also of interest. β-Hydroxybutyrate serves not only as an alternative fuel but also as a signaling molecule that inhibits the NLRP3 inflammasome and induces antioxidant gene expression (34, 71). Ketogenic diets and exogenous ketone supplementation have shown neuroprotective effects in neurodegenerative diseases, and their value in DR warrants further study.

## 6 Challenges and prospects for clinical translation

### 6.1 Early detection of NVU uncoupling before clinically visible retinopathy

NVU-oriented therapy emphasizes early intervention, yet current clinical diagnosis of DR still relies largely on funduscopic vascular changes. By the time overt retinopathy is evident, neuronal injury may already be irreversible. Sensitive methods for detecting early neurofunctional impairment are therefore essential (3). Electrophysiologic testing, such as multifocal electroretinography, can detect subclinical retinal dysfunction, with reduced amplitudes and prolonged implicit times indicating neuronal injury. However, routine use is limited by equipment requirements and time burden (75). Advances in OCT have enabled precise measurement of retinal layer thickness, and thinning of the ganglion cell complex may serve as an early marker of neurodegeneration (4). OCTA provides non-invasive visualization of the retinal microvasculature, allowing assessment of capillary nonperfusion and vascular density without dye injection (76).

Functional retinal imaging, including retinal oxygen saturation imaging and multispectral imaging, may help assess retinal metabolic status. Adaptive optics permits *in vivo* visualization of individual cone photoreceptors and capillaries, offering a means to detect cellular changes at an early stage (9). Biomarker discovery is also critical for early diagnosis and phenotyping (65). Proteomic and metabolomic studies of vitreous and aqueous humor have identified multiple molecules associated with DR severity, including VEGF, inflammatory mediators, oxidative stress markers, and miRNAs (27, 64). Blood-based biomarkers are more accessible, and extracellular vesicles, circulating miRNAs, and inflammatory factors are under active investigation. An ideal biomarker should predict disease risk, reflect key pathogenic processes, monitor treatment response, and support individualized therapy. The heterogeneity of DR underscores the need for precise phenotyping (10). In different patients, the dominant pathogenic process may vary, with inflammation, hypoxia, or neurodegeneration playing different roles (23, 29). Machine learning approaches based on multimodal data, including clinical, imaging, biomarker, and genotypic information, may enable more precise DR phenotyping and guide individualized treatment selection.

### 6.2 Delivering NVU-targeted therapies to the posterior segment

The unique anatomy of the eye poses major barriers to effective drug delivery (41). Systemic therapy is limited by the blood-retinal barrier and carries the risk of systemic adverse effects. Topical therapy is poorly suited for retinal disease because corneal and conjunctival barriers restrict posterior segment penetration, making it most useful for anterior segment disorders.

Intravitreal injection bypasses the BRB and remains the standard route for anti-VEGF and corticosteroid therapy. However, repeated injections increase the risk of infection, hemorrhage, and other complications, and long-term adherence is often suboptimal (38). Strategies to prolong drug action include long-acting formulations, sustained-release implants such as Ozurdex and Iluvien, and port delivery systems such as the ranibizumab port delivery system (41). Nanocarrier platforms, including liposomes, polymeric nanoparticles, and dendrimers, can improve drug stability and controlled release, and surface modification allows targeted delivery. Penetrating peptides and ligand modification, such as targeting VEGFR or transferrin receptors, may enhance cellular uptake and trans-BRB transport, thereby improving specificity and reducing off-target effects. Adeno-associated virus (AAV)-mediated gene therapy can achieve long-term expression, and a single intravitreal or subretinal injection may provide benefit for years. Different serotypes show distinct cellular tropism, with AAV2 preferentially transducing ganglion cells and Müller cells, whereas AAV8 and AAV9 have broader transduction profiles (13, 38). In DR, AAV-mediated delivery of antiangiogenic, neurotrophic, and anti-inflammatory factors remains in preclinical or early clinical development (77). Nonviral delivery systems are safer, but their efficiency and durability still need improvement. Cell therapy can be delivered by intravitreal injection, subretinal injection, or systemic infusion. Subretinal delivery provides stronger local targeting but is more invasive, whereas systemic mesenchymal stem cell (MSC) infusion depends on homing and has limited efficiency. In addition, ultrasound-mediated delivery, iontophoresis, and microneedle-based approaches are under investigation to achieve minimally invasive or non-invasive administration (41).

### 6.3 Stage-adapted combination therapy

A clinically useful NVU-based approach should not treat DR as a uniform vascular endpoint. The stage-adapted treatment concept proposed in this review is based on the idea that the dominant mechanism of NVU injury changes during disease progression. Early disease is more closely related to metabolic stress, oxidative injury, neuronal dysfunction, and glial maladaptation; intermediate disease is increasingly driven by inflammation, BRB instability, and vascular leakage; and advanced disease is characterized by ischemia, diabetic macular edema, and proliferative complications. Therefore, combination therapy should not be viewed as simply adding more treatments, but as matching therapeutic targets to the dominant biological process at each disease stage. Given the multifactorial pathogenesis of DR, single-target therapy is often insufficient, whereas multimodal interventions may produce additive or synergistic benefit (9, 10). Anti-VEGF therapy combined with corticosteroids is already used for refractory DME, targeting vascular permeability and inflammation, respectively (38). DRCR Protocol U showed that adding corticosteroids can provide additional benefit in some patients with an inadequate response to anti-VEGF therapy (41).

Combining anti-VEGF agents with neuroprotective therapies, such as BDNF, EPO, or citicoline, may address vascular and neuronal injury at the same time, although clinical evidence remains limited (3, 23, 72). Combined antioxidant and anti-inflammatory treatment may interrupt the cycle linking oxidative stress and inflammation. Combinations such as Nrf2 activators with NLRP3 inhibitors have shown synergistic protection in experimental models (30, 53). Metabolic regulation combined with targeted therapy reflects a comprehensive strategy that addresses both upstream causes and downstream manifestations. Intensive glycemic control remains fundamental, and future regimens may incorporate AGE inhibitors, sirtuin activators, and other metabolic modulators (32, 64). Natural compounds with antioxidant, anti-inflammatory, and neuroprotective properties may simplify combination therapy, but their efficacy and safety require rigorous clinical validation. More broadly, the clinical value of combination therapy depends on treatment timing and disease stage. In early disease, therapeutic emphasis may be placed on systemic metabolic control, antioxidant protection, neuroprotection, and preservation of glial homeostasis. In eyes with established vascular leakage or DME, anti-VEGF therapy, Ang-2/Tie-2-directed vascular stabilization, and corticosteroid-based anti-inflammatory treatment may be more clinically relevant. In advanced disease with ischemia, proliferative retinopathy, tractional complications, or non-clearing vitreous hemorrhage, multimodal or procedural interventions, including laser treatment, intravitreal therapy, and surgery, may be required (2, 38). This stage-adapted framework remains hypothesis-generating and should not be interpreted as a clinical guideline. Its value lies in organizing current and emerging therapies according to dominant disease biology. Future prospective studies are needed to validate the optimal timing, therapeutic combinations, patient selection, and long-term safety of these strategies.

### 6.4 Precision phenotyping and treatment selection

DR shows substantial interindividual variability. Some patients with long disease duration and poor glycemic control do not develop severe retinopathy, whereas others progress rapidly despite intensive management. This highlights the importance of genetic and host factors (1). Genome-wide association studies have identified several DR susceptibility loci involving angiogenesis, including VEGF and VEGFR; inflammation, including TNF-α and NOS3; oxidative stress, including SOD2 and CAT; and lipid metabolism, including APOE (9). These variants may influence disease susceptibility, progression rate, and treatment response. Pharmacogenomics examines how genetic variation affects drug response. Polymorphisms in VEGF have been associated with response to anti-VEGF therapy, and certain genotypes may predict better efficacy or a higher risk of resistance. Variants in metabolic enzyme genes can affect drug metabolism and clearance, while receptor gene variants may alter drug activity. Epigenetic markers, including DNA methylation patterns and miRNA profiles, may also serve as predictive biomarkers to guide therapy selection (55, 63, 65). For example, patients with a more inflammatory phenotype may derive greater benefit from anti-inflammatory treatment, whereas those with high VEGF expression may respond better to anti-VEGF therapy (62). Integrative multiomics analysis, including genomics, transcriptomics, proteomics, and metabolomics, can provide a more complete picture of individual disease biology. Artificial intelligence and machine learning may then identify patterns from large datasets, predict disease progression and treatment response, and support precision-based care.

### 6.5 Future directions: from mechanistic mapping to clinical translation

Although the NVU provides a useful framework for understanding DR pathogenesis, several key questions remain unresolved. Future research should move beyond isolated descriptions of neuronal, glial, or vascular injury and focus on how these components interact dynamically during the transition from early functional impairment to clinically visible retinopathy. In particular, four directions may be critical for translating the concept of NVU uncoupling into clinical practice.

First, single-cell and spatial multiomics approaches are needed to define cell-specific and region-specific changes within the diabetic retina. Single-cell RNA sequencing can identify transcriptional alterations in retinal neurons, Müller cells, microglia, astrocytes, endothelial cells, and pericytes, whereas spatial transcriptomics can preserve tissue architecture and reveal ligand-receptor interactions within the NVU (9, 10). Integration of transcriptomics, proteomics, metabolomics, lipidomics, and epigenomics may help construct a more complete map of neurovascular crosstalk under diabetic conditions. Such approaches may identify which cell populations initiate NVU uncoupling, which signaling pathways sustain disease progression, and which molecular nodes are most suitable for therapeutic targeting.

Second, improved functional imaging and biomarker strategies are required to detect NVU dysfunction before irreversible structural damage occurs. Current clinical diagnosis of DR still depends largely on visible vascular lesions, but neurofunctional impairment and subtle microvascular dysregulation may occur earlier (3, 4). Multifocal electroretinography, OCT, OCTA, retinal oxygen saturation imaging, adaptive optics, and functional retinal imaging may provide complementary information on neuronal activity, retinal layer integrity, capillary perfusion, oxygen metabolism, and cellular-level structural changes (9, 75). In parallel, circulating or intraocular biomarkers, including inflammatory mediators, oxidative stress markers, miRNAs, extracellular vesicles, and metabolomic signatures, may help identify patients with early NVU impairment (64, 65). Combining multimodal imaging with molecular biomarkers may support a more precise definition of preclinical or early-stage DR.

Third, future therapeutic development should focus on stage-adapted combination strategies rather than single-pathway intervention. In the early stage of diabetes, treatment may need to prioritize systemic metabolic control, antioxidant defense, neuroprotection, and preservation of glial homeostasis. In intermediate disease, anti-inflammatory and vascular-stabilizing approaches may be required to limit BRB breakdown and prevent progression to diabetic macular edema. In advanced disease, anti-VEGF therapy, Ang-2/Tie-2 modulation, corticosteroids, laser treatment, or surgery may still be necessary to control macular edema, ischemia, or proliferative complications (38, 41, 61). A clinically useful NVU-based strategy should therefore match therapeutic targets to the dominant pathogenic process in each patient, such as neurodegeneration, inflammation, vascular leakage, ischemia, or metabolic vulnerability.

Fourth, more human-relevant disease models and rigorous clinical validation are essential. Current diabetic animal models do not fully reproduce the chronic course, cellular complexity, and treatment heterogeneity of human DR. Retinal organoids, human induced pluripotent stem cell-derived retinal cells, organ-on-chip systems, and co-culture models incorporating neurons, glial cells, endothelial cells, and pericytes may provide more reliable platforms for studying NVU interactions and testing therapeutic candidates. However, findings from these models must ultimately be validated in well-designed clinical studies with standardized imaging, functional endpoints, biomarker assessment, and sufficiently long follow-up.

Several emerging areas, including the retina-brain axis, sex-related differences, gut microbiome, and artificial intelligence, may further refine the understanding and management of DR. These factors should be integrated into future studies when they directly inform NVU biology, risk stratification, or treatment response. In particular, artificial intelligence may help combine clinical data, imaging features, biomarkers, and multiomics profiles to identify disease subtypes and predict progression. Ultimately, future research should aim to transform NVU uncoupling from a mechanistic concept into a clinically actionable framework for early diagnosis, precise phenotyping, and individualized treatment in DR.

## 7 Conclusion

Diabetic retinopathy should be viewed as a diabetes-driven neurovascular degenerative complication rather than a purely microvascular endpoint. Chronic hyperglycemia, oxidative stress, inflammation, VEGF/Ang-Tie imbalance, impaired intercellular communication, and epigenetic memory converge to disrupt coordinated interactions among retinal neurons, glial cells, endothelial cells, pericytes, and extracellular matrix components. This process leads to early NVU uncoupling, blood-retinal barrier breakdown, retinal dysfunction, and ultimately vision-threatening diabetic retinal complications. Current therapies, including anti-VEGF agents, corticosteroids, and Ang-2/Tie-2-directed therapy, have substantially improved the management of DME and proliferative DR. However, most established treatments mainly address vascular leakage, inflammation, or angiogenesis after clinically apparent disease has developed. A broader NVU-based framework should integrate systemic metabolic control, neuroprotection, anti-inflammatory modulation, vascular stabilization, and restoration of intercellular signaling. Future studies should define reliable biomarkers of early NVU uncoupling, stratify patients according to dominant molecular mechanisms, and validate stage-adapted combination therapies in well-designed clinical studies. Such a shift from late vascular rescue to early integrated neurovascular protection may provide a more precise strategy for preventing vision loss in diabetes.
